# The Effect of Comorbidity between Tinnitus and Dizziness on Perceived Handicap, Psychological Distress, and Quality of Life

**DOI:** 10.3389/fneur.2017.00722

**Published:** 2017-12-22

**Authors:** Masatoshi Miura, Fumiyuki Goto, Yozo Inagaki, Yasuyuki Nomura, Takeshi Oshima, Nagisa Sugaya

**Affiliations:** ^1^Department of Otolaryngology-Head and Neck Surgery, Nihon University School of Medicine, Itabashi, Tokyo, Japan; ^2^Department of Otolaryngology, National Hospital Organization Tokyo Medical Center, Tokyo, Japan; ^3^Department of Otolaryngology, Hino Municipal Hospital, Tokyo, Japan; ^4^Unit of Public Health and Preventive Medicine, School of Medicine, Yokohama City University, Yokohama, Japan

**Keywords:** tinnitus, dizziness, Dizziness Handicap Inventory, psychological distress, quality of life

## Abstract

Tinnitus and dizziness are common complaints encountered in the department of otolaryngology. We hypothesized that when patients complain of both tinnitus and dizziness, perceived handicap, impairment of quality of life, and emotional distress are more severe than the patient who complain of either tinnitus or dizziness. The subjects for this study were 736 patients who visited Hino Municipal Hospital between August 2010 and March 2012, complaining of tinnitus or dizziness. The subjects were divided into three groups depending upon their chief complaints—group B had patients with both tinnitus and dizziness (*N* = 75), group T had patients with tinnitus (*N* = 145), and group D had patients with dizziness (*N* = 516). Assessments were performed using Tinnitus Handicap Inventory (THI) for groups B and T, Dizziness Handicap Inventory (DHI) for groups B and D, Medical Outcomes Study 8-items Short-Form Health Survey (SF-8), and Hospital Anxiety and Depression Scale (HADS). The THI score of group B was higher than that of group T. The scores of PCS (physical component of SF-8) of groups B and D were lower than that of group T. However, there were no significant differences in the DHI scores of groups B and D, and the HADS scores of the three groups. While the physical quality of life was found to vary depending on the presence of dizziness in patients with tinnitus, it was not found to vary depending on the presence of tinnitus in patients with dizziness. It is therefore important to consider the functional impact resulting from dizziness in patients with tinnitus.

## Introduction

Tinnitus and dizziness are common complaints encountered in the department of otolaryngology. Although no serious hidden diseases in are usually found in these patients, their quality of life (QOL) is sometimes markedly impaired. For many years, hearing loss has been understood to be the most common cause of tinnitus (1), and about 40% of patients cannot identify any cause associated with tinnitus onset (2). Tinnitus is frequently associated with sensorineural hearing loss and sometimes with dizziness or vertigo (3). It is thus necessary for the same physician to evaluate and treat all physical and psychological complaints of the patients. However, in very specific healthcare systems, patients with tinnitus are treated in the auditory department and patients with dizziness are treated in the vestibular department.

It is easy to comprehend that the functional impact resulting from tinnitus is influenced by a variety of physical and psychological conditions. The comorbidities such as hyperacousis (4), hearing loss (5, 6), insomnia (7, 8), depression (9, 10), headache (11, 12), and pain syndromes (13) play a major role for tinnitus-related impairment in QOL. Tinnitus-related health burden can be measured by specific validated tinnitus questionnaires like the Tinnitus Handicap Inventory (THI) (14). Thus, we are interested in not only tinnitus itself, but also other physical conditions that influence the functional impact due to tinnitus.

Otologic disorders are the most common cause of subjective tinnitus (15). Most cases of tinnitus result from the same conditions that cause hearing loss (15). In some disorders, like Meniere’s disease, and some of sudden deafness patients complain not only tinnitus bus also dizziness. In these disorders, a common mechanism or a strong relationship may exist between tinnitus and dizziness. The mood and QOL resulting from this physical distress may be affected by the presence or absence of either of the symptoms.

However, only a few studies on the relationship between dizziness and tinnitus have been reported. Stephens and Hallam (16) found that patients with tinnitus experiencing dizziness had elevated somatic anxiety and phobic anxiety. These reports mainly focused not on the handicap due to dizziness itself but on the psychosocial factors related to dizziness.

We hypothesized that the impact of tinnitus or dizziness, psychological distress, and the impairment of QOL are more severe in patients who complain of both tinnitus and dizziness than in those who complain of either tinnitus or dizziness.

## Materials and Methods

### Participants

The participants of the present study were patients who visited Hino Municipal Hospital between August 2010 and March 2012 complaining of tinnitus or dizziness. The participants were divided into three groups depending on the chief complaints on their initial visits—group B had patients with both tinnitus and dizziness, group T had patients with tinnitus alone, and group D had patients with dizziness alone.

The diagnosis of vestibular disorders was based on the diagnostic criteria defined by the Barany Society Classification Committee (17–22). With respect to psychogenic diagnosis, the psychogenic vertigo was considered when the patient’s dizziness cannot be attributed to other vestibular disorders and the patient was suffered from psychiatric disorders (23). Psychogenic tinnitus was considered when the patient’s tinnitus cannot be attributed to otologic disorders and the patient was suffered from psychiatric disorders (24).

All required ethical approvals were obtained from the Institutional Review Board at the Hino Municipal Hospital (No. 29-5). The study was, therefore, performed in accordance with the ethical standards laid down by the 1964 Declaration of Helsinki and its amendments. We obtained the written informed consent from the participants of this study.

### Measures

#### Japanese Version of the THI

The Japanese version of the THI (14) is a 25-item questionnaire that quantifies the impact of tinnitus. The total score ranges from 0 (no disability) to 100 (severe disability).

#### Japanese Version of the Dizziness Handicap Inventory

The Japanese version of the Dizziness Handicap Inventory (DHI) (5, 25) is a 25-item questionnaire that is useful for documenting the consequences of vestibular and/or cochlear impairment. The total score ranges from 0 (no disability) to 100 (severe disability).

#### Hospital Anxiety and Depression Scale–Anxiety Scale, Japanese Version

The Hospital Anxiety and Depression Scale (HADS) (7, 26) is a self-reported questionnaire consisting of 14 questions. It has anxiety and depression subscales, each with seven items. This psychometric instrument was chosen because all its items refer solely to an emotional state, and do not consider somaticsymptoms.

#### Japanese Version of Medical Outcomes Study 8-Item Short-Form Health Survey

Health-related QOL was evaluated using the Japanese version of the Medical Outcomes Study 8-Item Short-Form Health Survey (SF-8) questionnaire; the validity and reliability of which have already been confirmed (9, 27). SF-8 comprises of eight items: physical functioning, role limitation due to physical problems, body pain, general health, vitality, social functioning, role limitation due to emotional problems, and mental health. The physical health component summary score (PCS) and mental health component summary score (MCS) were measured using the Norm-Based Scoring method, which is based on a large-scale population study conducted in Japan (28). Higher scores on these subscales indicate better health-related QOL.

### Procedure

Depending on the chief complaints, a set of questionnaires were used for the evaluation. HADS and SF-8 were used for all patients. For the patients in group T, THI is added; for those in group D, DHI is added; and for the patients in group B, both THI and DHI were added.

### Statistical Analysis

Data analysis was performed using IBM SPSS Statistics 22.0 software. The χ^2^ test was used to compare the sex ratio between the groups. The prevalence of psychogenic tinnitus and/or dizziness was compared between groups using the χ^2^ test because the prevalence is possible to affect psychological indexes. Comparisons between groups in terms of the scores of the DHI and the THI were performed using *t*-test. Group differences in HADS and SF-8 were measured using two-way analysis of variance (ANOVA). *Post-hoc* comparisons between the groups were made using Bonferroni’s multiple comparison test. Pearson’s correlation analysis was then performed for each group to confirm any relationships between the variables. The significance level was set at *p* < 0.05.

## Results

### Characteristics of the Participants

There were 867 patients who met the listed requirements. We further excluded those who had missing THI and DHI data (*N* = 131). Thus, 736 patients (271 male and 465 female patients, mean age = 56.15 ± 18.06 years) remained in the analysis. Some patients did not provide any data regarding the HADS anxiety (HADS-A; valid data: *N* = 723), HADS depression (HADS-D; valid data: *N* = 722), and SF-8 (valid data: *N* = 412). Table 1 shows the diagnoses of the participants according to the history taken on their initial visit. In group B, the most common diagnosis was Meniere’s disease followed by psychogenic. In group T, the most common diagnosis was other or unknown followed by sudden deafness. Ii is usually difficult to define the cause of tinnitus in patients with only tinnitus. In group D, the most common diagnosis was Benign Paroxysmal Positional Vertigo. The χ^2^ test of the prevalence of psychogenic tinnitus and/or dizziness indicated no significant difference between groups. ANOVA of age indicated significant group differences (*F* [2, 733] = 3.42, *p* = 0.03), and *post-hoc* analysis showed that group T had an older age than group D (*p* = 0.03). The χ^2^ test of the sex ratio indicated that group T had a significantly higher ratio of male patients and lower ratio of female patients (χ^2^ [2] = 12.8, *p* = 0.002). Correlation analysis between age and each of the other variables showed statistically significant but weak correlation between age and HADS-A (*r* = −0.18, *p* < 0.001); however, age was not found to be significantly correlated with the other variables. Female participants had higher DHI (*t* [589] = 2.96, *p* = 0.003) and HADS-A (*t* [721] = 2.69, *p* = 0.007) scores than the male participants.

**Table 1:** Primary diagnoses of the participants.

	Group B	Group T	Group D
BPPV	4		166
Psychogenic	6	7	35
Unilateral vestibulopathy			56
Vestibular neuritis	4		36
Meniere’s disease	11	5	34
Vestibular migraine	6		32
Sudden deafness or sequelae of sudden deafness	9	25	20
Orthostatic dysfunction			10
Recurrent vestibulopathy	1		9
Bilateral vestibulopathy			8
Head trauma	1	3	8
Presbystasis	1		8
Neurovascular compression			6
Other or unknown	32	105	88
Total	75	145	516

*BPPV, Benign Paroxysmal Positional Vertigo*.

### Comparison between THI, DHI, SF-8, and HADS

The THI score for group B was significantly higher than that for group T (*t* [218] = 2.22, *p* = 0.03) (see Table 2). ANOVA of the SF-8 scores showed significant group differences in the score of PCS (*F* [2, 409] = 9.60, *p* < 0.001). *Post-hoc* test indicated that the scores of PCS in group B (*p* = 0.002) and group D (*p* < 0.001) were significantly lower than those in group T. However, there was no significant difference in the DHI scores of groups B and D and in the subscores of HADS between the three groups.

**Table 2:** Sex ratio, age, and scores of THI, DHI, HADS, and SF-8 in each group.

	Group B	Group T	Group D
Male/female	25/50	72/73	174/342
Age	55.23 ± 16.23	59.66 ± 17.50	55.30 ± 18.37
THI	31.89 ± 24.83	24.62 ± 22.06	−
DHI	31.09 ± 23.45	−	35.09 ± 24.57
HADS-A	7.15 ± 4.77	5.79 ± 4.06	6.41 ± 4.28
HADS-D	5.80 ± 3.89	5.02 ± 3.57	5.55 ± 3.67
SF-8 PCS	44.40 ± 8.10	49.18 ± 6.58	45.38 ± 8.01
SF-8 MCS	45.60 ± 9.31	47.87 ± 7.36	46.34 ± 8.65

*THI, Tinnitus Handicap Inventory; DHI, Dizziness Handicap Inventory; HADS-A and HADS-D, Hospital Anxiety and Depression Scale–anxiety scale and depression scale; SF-8 PCS and MCS, Medical Outcomes Study 8-Items Short-Form Health Survey–physical health component summary score and mental health component summary score*.

### Correlation between Variables in Each Group

In group B, significant correlations were observed between the scores of THI and DHI (*r* = 0.28, *p* = 0.01), subscores of HADS (HADS-A: *r* = 0.38, *p* < 0.001, HADS-D: *r* = 0.33, *p* = 0.004), or the MCS score (*r* = −0.32, *p* = 0.03), and between the DHI and HADS-A scores (*r* = 0.27, *p* = 0.02) or the PCS score (*r* = −0.36, *p* = 0.01) (see Table 3).

**Table 3:** Correlations between the variables in each group.

Group B	THI	DHI	HADS-A	HADS-D	SF-8 PCS	SF-8 MCS
THI	–	0.28*	0.38***	0.33**	−0.22	−0.32*
DHI	0.28*	–	0.27*	0.11	−0.36*	−0.17
**Group T**	**HADS-A**	**HADS-D**	**SF-8 PCS**	**SF-8 MCS**		
THI	0.35***	0.27**	−0.33**	−0.50***		
**Group D**	**HADS-A**	**HADS-D**	**SF-8 PCS**	**SF-8 MCS**		
DHI	0.32***	0.28***	−0.31***	−0.30***		

*p < 0.05.

**p < 0.01.

***p < 0.001.

*THI, Tinnitus Handicap Inventory; DHI, Dizziness Handicap Inventory; HADS-A and HADS-D, Hospital Anxiety and Depression Scale–anxiety scale and depression scale; SF-8 PCS and MCS, Medical Outcomes Study 8-Items Short-Form Health Survey–physical health component summary score and mental health component summary score*.

In group T, there were significant correlations between THI and the subscores of HADS (HADS-A: *r* = 0.35, *p* < 0.001, HADS-D: *r* = 0.27, *p* = 0.001) and SF-8 (PCS: *r* = −0.33, *p* = 0.001, MCS: *r* = −0.50, *p* < 0.001).

In group D, there were significant correlations between DHI and the subscores of HADS (HADS-A: *r* = 0.32, *p* < 0.001, HADS-D: *r* = 0.28, *p* < 0.001) and SF-8 (PCS: *r* = −0.31, *p* < 0.001, MCS: *r* = −0.30, *p* < 0.001).

## Discussion

The handicap resulting from tinnitus was found to be exacerbated in patients with both tinnitus and dizziness, although the handicap resulting from dizziness did not differ in patients with dizziness and tinnitus. Physical health-related QOL may be compromised by the presence of dizziness rather than by the presence of both tinnitus and dizziness. Emotional distress and mental health-related QOL were not found to differ between groups. While the handicap related to tinnitus or dizziness was found to be correlated to other variables in all patient groups, there was no significant correlation between the handicap related to tinnitus and physical health-related QOL and between the handicap relating to dizziness and anxiety or mental health-related QOL in patients with both tinnitus and dizziness. Decreased physical health-related QOL in patients with both tinnitus and dizziness may be associated with not the handicap related to tinnitus but to the presence of dizziness. Tinnitus is perceived both during rest and during physical activity while dizziness occurs mainly during physical activity. Thus, each of the symptoms can affect the physical health-related QOL and result in the differences between the groups.

Although the scores of the some of the questionnaires were statistically deference among groups, the interpretation of the obtained results required special caution. For example, the THI values of groups B and T were both within the range of mild handicap. Also the score of SF-8 of each group was close to 50, which are close to standard values.

Regarding the prevalence of psychogenic tinnitus and/or dizziness, there was no significant difference between groups, and thus the prevalence of psychogenic tinnitus and/or dizziness may not affect the results of the present study. While older age and greater ratio of women participants were observed among the patients with tinnitus in our study, the correlation between age and anxiety was found to be negative, although female participants showed higher levels of anxiety and the handicap resulting from dizziness. However, age and sex ratio are unlikely to be confounders in the present study for the following reasons: (1) there was no difference in the sex ratio between groups B and D, and thus the sex ratio did not affect the DHI score among the groups, (2) the THI score and the PCS score did not significantly correlate with age, and (3) there was no significant difference in the HADS-A score of the groups. The results of this study suggest the need to confirm the presence of symptoms of dizziness cautiously in patients with tinnitus and to assess the effect of the handicap resulting from dizziness on their QOL.

In routine practice, physicians tend to deal with only one chief complaint of the patients, even if the patients have multiple complaints. One reason for this is the current medical system. In very specific healthcare systems, the specialties of the physicians are restricted; for instance, a physician may be specialized in only dizziness or tinnitus. Physicians have to deal with both tinnitus and dizziness in patients with Meniere’s disease, in which hearing loss, tinnitus, and dizziness are included in the diagnostic criteria. Since tinnitus and dizziness are common complaints, physicians have not paid much attention to the coexistence of these complains; instead, physicians tend to focus on only one.

The present study had several limitations. First, we did not consider main complaints other than dizziness or tinnitus and underlying diseases. Second, the results of present study could be affected by the presence of Meniere’s disease or dizziness associated with sudden deafness in many participants in group B. These diseases tend to be more severe than other diseases associated with dizziness or tinnitus. Third, we did not consider the duration and symptoms type of the diseases. The symptoms of the participants in the earlier stages of the disease were probably more severe and there would be deference between episodic and chronic disorders. In future, we should control the duration of the disease or compare between episodic and chronic vestibular disorders. Fourth, we conducted a cross-sectional study and a prospective cohort study, would be required instead, to investigate the therapeutic change in dizziness and tinnitus. Fifth there is not enough data about the tinnitus in a wide range of vestibular diagnoses. Sixth, it would be interesting to analyze the relationship between tinnitus and hearing loss (6). However, the hearing loss threshold was not available in this study and the relationship between tinnitus and hearing loss could not be determined, but it will be investigated in future studies.

## Conclusion

The handicap resulting from tinnitus was exacerbated by the coexistence of dizziness but the reverse was not found. The functional impact of dizziness was similar in those having dizziness alone or together with tinnitus. The results of this study suggest the need to confirm the presence of symptoms of dizziness cautiously in patients with tinnitus and to assess the effect of the handicap resulting from dizziness on their QOL.

## Ethics Statement

All required ethical approvals were obtained from the Institutional Review Board at the Hino Municipal Hospital. The study was, therefore, performed in accordance with the ethical standards laid down by the 1964 Declaration of Helsinki and its amendments.

## Author Contributions

MM and NS contributed to the analysis and interpretation of the data. FG contributed to the concept and design of the work, and analysis and interpretation of the data. YI, YN, and TO contributed to the concept and design of the work. All the authors approved the final version of the manuscript and agreed to be accountable to all aspects of this work.

## Conflict of Interest Statement

The part of the study is supported by a grant from the Mental Health Okamoto 2017.
